# Bonding performance of universal adhesive systems with dual-polymerising resin cements to various dental substrates: in vitro study

**DOI:** 10.1186/s12903-025-05438-z

**Published:** 2025-01-20

**Authors:** Shunsuke Takano, Rena Takahashi, Tomoko Tabata, Chen Zeng, Masaomi Ikeda, Yasushi Shimada

**Affiliations:** 1 Department of Cariology and Operative Dentistry, Graduate School of Medical and Dental Sciences, Institute of Science Tokyo, 1−5−45 Yushima, Bunkyo−Ku, Tokyo, 113−8549 Japan; 2 Department of Oral Biomedical Engineering, Graduate School of Medical and Dental Sciences, Institute of Science Tokyo, 1−5−45 Yushima, Bunkyo−Ku, Tokyo, 113−8549 Japan

**Keywords:** Universal adhesive, Dual-polymerising resin cement, Micro-shear bond strength, Enamel, Dentin, Zirconia, Lithium disilicate ceramics, Resin block

## Abstract

**Background:**

Resin cements often require substrate-specific pretreatment. Recently, universal adhesive systems have been introduced, simplifying procedures by eliminating the need for multiple adhesives and offering options that do not require light curing. This study investigated the bonding performance of universal adhesive systems combined with dual-polymerising resin cements on enamel, dentin, zirconia, lithium disilicate ceramics (LDS), and resin blocks.

**Methods:**

Two universal adhesive and dual-polymerising resin cement combinations from the same manufacturer were tested: Bondmer Lightless II (BLII) with Estecem II (ECII), and Scotchbond Universal Plus adhesive (SBU) with RelyX Universal resin cement (RXU). Enamel, dentin, zirconia (Katana Zirconia UTML), LDS (IPS e.max CAD CEREC), and resin blocks (Katana Avencia P Block) were used as substrates. The universal adhesive was applied to all bonding surfaces, followed by resin cement application in micro-bore Tygon tubes and light curing for 40 s. Micro-shear bond strength (μSBS) was measured after 0 thermal cycles (0TC) or 10,000 thermal cycles (10kTC) (*n* = 20). Statistical analyses were conducted using t-tests and Welch's t-tests with Bonferroni correction (*α* = 0.05), and failure modes were examined.

**Results:**

In the BLII/ECII group, the mean μSBS values exceeded 15 MPa for all substrates at 0TC. After thermocycling, μSBS increased significantly for the enamel (*p* < 0.05), remained unchanged for dentin and zirconia (*p* > 0.05) and decreased for LDS and resin blocks (*p* < 0.05). In the SBU/RXU group, 0TC μSBS values exceeded 15 MPa for enamel, zirconia, and resin blocks, but thermocycling significantly decreased μSBS for all substrates (*p* < 0.05). Comparison between BLII/ECII and SBU/RXU group showed no significant differences for enamel and resin blocks at 0TC (*p* > 0.05), but the BLII/ECII group exhibited higher SBS in the other groups (*p* < 0.05). Adhesive failure was the most frequently observed failure type across all groups.

**Conclusion:**

The adhesive performance on diverse dental substrates including enamel, dentin, zirconia, LDS, and resin blocks was notably affected by the selection of universal adhesive systems in combination with dual-polymerising resin cements that were applied. The BLII/ECII combination demonstrated long-term stable bonding performance for enamel, dentin, and zirconia.

## Introduction

Resin cements have revolutionised modern dentistry owing to its unparalleled adhesion and durability compared to traditional cements [1]. These cements exhibit exceptional bond strength, resistance to microleakage, and the ability to reduce the risk of secondary caries while extending the lifespan of restorations [2]. Additionally, their aesthetic properties, such as translucency and the ability to match various tooth shades, make resin cements an indispensable material for achieving both functional and visually pleasing results in restorative procedures [3]. However, most resin cements require pretreatment of tooth structure and dental materials.

To address this complexity, adhesive systems classified as ‘universal’ adhesives, designed for both direct and indirect restorations, have recently been introduced in the dental market [4–6]. These universal adhesive systems simplify operative procedures by reducing steps and eliminating the need for different adhesives for various substrates. Many universal adhesive systems contain several functional monomers that can react with tooth substrates and inorganic dental material surfaces [7]. Recently, a universal adhesive system that does not require light curing has been introduced [8]. This feature allows for more reliable bonding in areas where light penetration is challenging, such as in deep restorations. Additionally, it simplifies the clinical procedure by reducing the need for precise light curing.

Universal adhesives are often recommended to be used in combination with specific resin cements. Among these dual-polymerizing resin cements, there are those without acidic functional monomers, such as Estecem II (ECII: Tokuyama Dental, Tokyo, Japan), and those with acidic functional monomers, such as RelyX Universal Resin Cement (RXU: 3 M ESPE, St. Paul, MN, USA). However, there is limited information available on the bonding efficacy of universal adhesive systems and compatible dual-polymerising resin cements across diverse substrates, especially on the long-term prognosis.

Therefore, this study examined the bonding efficacy of universal adhesive systems with dual-polymerising resin cements on enamel, dentin, zirconia, lithium disilicate ceramics (LDS) and resin block. This study proposed the following null hypotheses: (i) the choice of universal adhesive systems with dual-polymerising resin cements would not affect the bonding efficacy to various substrates, and (ii) thermocycling would not affect the bonding efficacy of universal adhesive systems with dual-polymerising resin cements.

## Materials and methods

### Specimen preparations

**Fig. 1:**
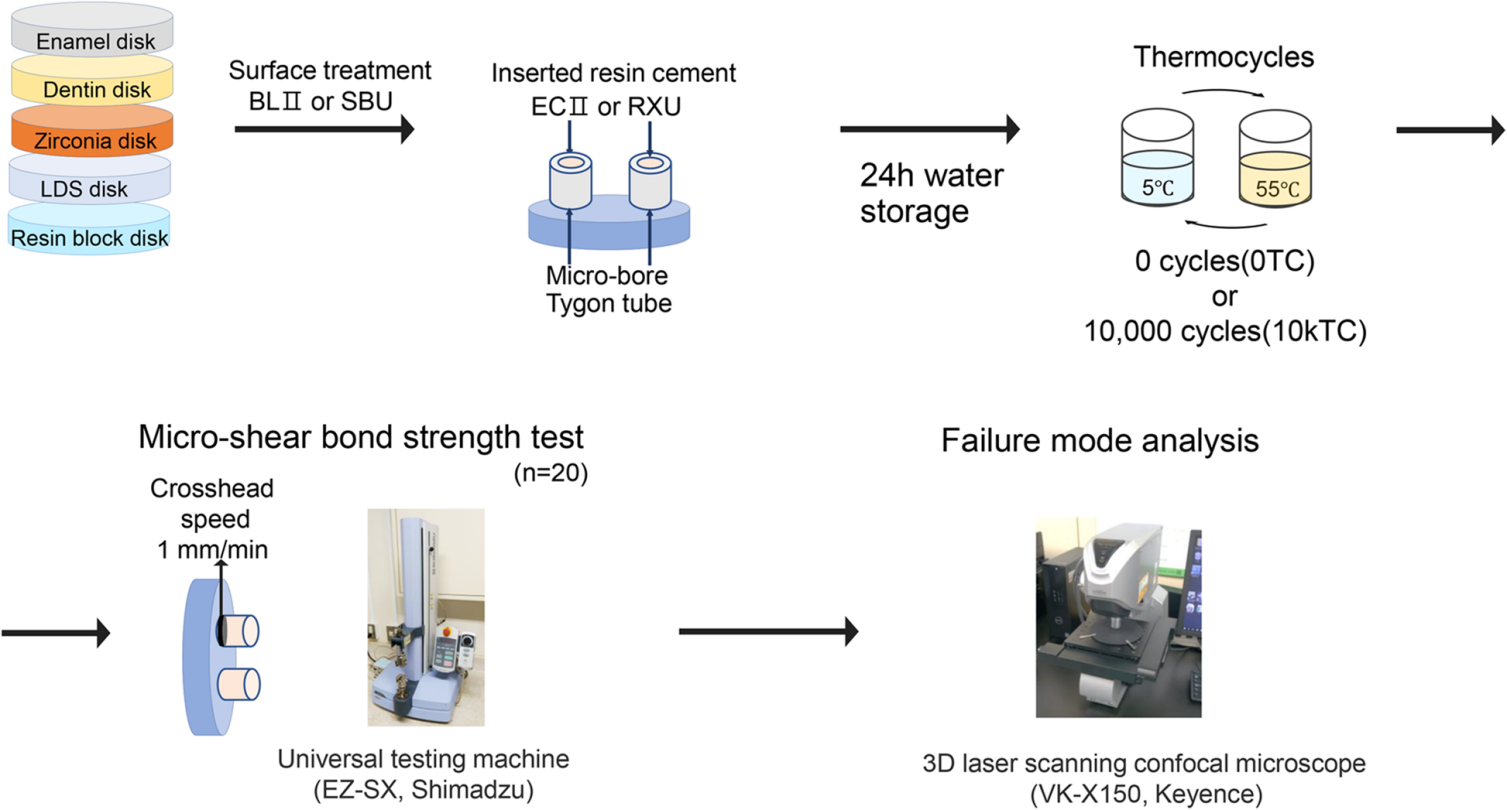
Schematic representation of the specimen preparation process

The study design was based on previously published research [8, 9]. Figure 1 presents a schematic of the specimen preparation process. The materials used in this study are detailed in Table 1. The Ethics Committee of the Graduate School and Hospital at Tokyo Medical and Dental University (No. D2013-022–06) approved the study protocol. Twenty-eight extracted caries-free human molars were stored in a 0.1% thymol solution at 4 °C until the experiments commenced. The roots were removed from the dental specimens, and the crowns were bisected along the mesio-distal axis. This sectioning process was accomplished using a diamond-tipped saw (Isomet, Buehler, Lake Bluff, IL, USA). Following this, enamel and dentin specimens with approximate dimensions of 2 mm thickness, 5 mm width, and 10 mm height were prepared. These were ground with 600-grit silicon carbide paper to produce a homogeneous smear layer.

**Table 1:** Materials used in this study

Material	Compositions	Application instructions	Batch No
Bondmer Lightless II Code: BLII (Tokuyama Dental, Tokyo, Japan)	Bottle A: Phosphate acid monomer, MTU-6, Bis-GMA, TEGDMA, HEMA, acetone, etc	Mix one drop of Bottle A and Bottle B Apply to the sample for 10 s, then air dry	2U0035
	Bottle B: γ-MPTES, acetone, ethanol, water, borate catalyst, peroxide, etc		
Estecem II Code: ECII Universal shade (Tokuyama Dental)	Paste A: Silica zirconia filler, Bis-GMA, TEGDMA, Bis-MPEPP, etc	The tygon tube was injected with cement, lined up on the sample surface, and light irradiated for 40 s	2P0020
	Paste B: Silica zirconia filler, Bis-GMA, TEGDMA, Bis-MPEPP peroxide, CQ		
Scotchbond Universal Plus Adhesive Code: SBU (3M ESPE, St. Paul, MN, USA)	2-propenoic acid 2-methyl- diesters with 4,6-dibromo-1,3-benzenediol 2-(2-hydroxyethoxy)ethyl 3-hydroxypropyl diethers, HEMA, 2-propenoic acid, 2-methyl-, 3-(triethoxysilyl)propyl ester (MPTES), (3-aminopropyl) triethoxysilane (APTES) and reaction products with vitreous silica, 2-propenoic acid 2-methyl- reaction products with 1,10-decanediol and phosphorus oxide (P_2_O_5_) (10-MDP), water, ethanol, camphorquinone, copolymer of acrylic and itaconic acid, N,N-dimethylbenzocaine, acetic acid, copper(2 +) salt, monohydrate pH = 2.7	Apply to the sample for 20 s, then air dry	642539
RelyX Universal Resin Cement Code: RXU A1 shade (3M ESPE)	Base: 2-propenoic acid, 2-methyl-, 3(trimethoxysilyl) propyl ester, reaction products with vitreous silica, diurethanedimethacrylate, triethylene glycol dimethacrylate, mixture of mono-di-and tri-glycerol dimethacrylate ester of phosphoric acid, silane, trimethoxyoctyl-, hydrolysis products with silica, t-amyl hydroperoxide, 2,6-di-tert-butyl-p-cresol, 2-hydroxyethyl methacrylate, Methyl Methacrylate, Acetic acid, copper(2 +) salt, monohydrate	The tygon tube was injected with cement, lined up on the sample surface, and light irradiated for 40 s	628163
	Cataryst: diurethanedimethacrylate, ytterbium(III) fluoride, glass powder(65,997–17-3), surface modified with 2-propenoic acid, 2 methyl-.3- (trimethoxysilyl)propyl ester(2530–85-0) and phenyltrimethoxy silane (2996–92-1), bulk material, triethylene glycol dimethacrylate,silane, trimethoxyoctyl-, hydrolysis product with silica, L-Ascorbic acid,6-hexadecanoate, hydrate(1:2), Titanium dioxide, triphenyl phosphite, 2-hydroxyethyl methacrylate, N,N-dimethylbenzocaine		
Katana Zirconia UTML Code: zirconia A2 shade (Kuraray Noritake Dental, Tokyo, Japan)	Zirconium dioxide, yttrium trioxide, hafnium dioxide, inorganic pigment, organic binder	The surface was subjected to 50 μm alumina sandblast at 0.2 MPa for 60 s from a distance of 10 mm, followed by ultrasonic cleaning with distilled water for 2 min	204
IPS e.max CAD CEREC/inLab Code: LDS LT A2 C14 (Ivoclar Vivadent, Schaan, Liechtenstein)	Silicon dioxide, lithium oxide, potassium oxide, phosphorus pentoxide, zirconium oxide, zinc oxide, aluminum oxide, magnesium oxide, cerium oxide, etc	The surface was air-abraded with 50 μm alumina particles at 0.2 MPa for 60 s from a distance of 10 mm, followed by ultrasonic cleaning with distilled water for 2 min	CA0011
Katana Avencia P Block Code: resin block 14/A2LT (Kuraray Noritake Dental)	Barium glass, silica, methacrylic acid monomer mixed filler, methacrylic acid monomer (UDMA), colorants, etc	The surface was air-abraded with 50 μm alumina particles at 0.2 MPa for 60 s from a distance of 10 mm, followed by ultrasonic cleaning with distilled water for 2 min	BP0053

The composition listed is the information provided by the manufacturers

*Bis-GMA* Bisphenol-A-diglycidylmethacrylate, *Bis-MPEPP* 2,2'-bis (4-methacryloxy polyethoxyphenyl) propane, *CQ* Camphorquinone, *HEMA* Hydroxyethyl methacrylate, *10-MDP* 10-methacryloyloxydecyl dihydrogen phosphate, *MTU-6* 6-methacryloxyhexyl 2-thiouracil-5-carboxylate, *γ-MPTES* γ-mercaptopropyl trimethoxysilane, *TEGDMA* Triethylene glycol dimethacrylate, *UDMA* Urethane-dimethacrylate monomer-1,6-bis-[methacryloyloxy-2-ethoxycarbonylamino]

Fully sintered zirconia disk-shaped specimens with approximate dimensions of 5 mm thickness and 12 mm diameter by sintering at 1550 °C for 2 h in a sintering furnace (Esthemat SlimII, Shofu, Kyoto, Japan) were obtained from Katana Zirconia UTML (Kuraray Noritake Dental, Tokyo, Japan). LDS specimens with approximate dimensions of 2 mm thickness, 15 mm width, and 15 mm length were prepared using IPS e.max CAD CEREC/in Lab (Ivoclar Vivadent, Schaan, Liechtenstein) using a diamond-tipped saw (Isomet). These LDS specimens underwent a crystallisation-firing process in a furnace (Programat Ep3010, Ivoclar Vivadent) at 830 °C for 7 s, followed by 850 °C for 10 min under vacuum. Resin block specimens with approximate dimensions of 2 mm thickness, 15 mm width, and 15 mm length were fabricated from Katana Avencia P Block (Kuraray Noritake Dental) using a diamond-tipped saw (Isomet). Specimens of zirconia, LDS, and resin block were subjected to 600-grit silicon carbide paper under running water and air-abraded with 50 μm alumina particles (Cobra, Renfert, Hilzingen, Germany) at 0.2 MPa for 60 s from a distance of 10 mm, using a blasting machine (Basic eco, Renfert). They were then ultrasonically cleaned in water for 2 min.

### Microshear bond strength (μSBS) test

Two universal adhesive systems from the same manufacturers, Bondmer Lightless II (BLII: Tokuyama Dental) and Scotchbond Universal Plus Adhesive (SBU: 3 M ESPE), in combination with the dual-polymerising resin cements ECII and RXU, respectively, were evaluated.

The enamel, dentin, zirconia, LDS, and resin block specimens were treated with either BLII or SBU according to the manufacturers' guidelines (refer to Table 1). Micro-bore Tygon tubes (Saint-Gobain Performance Plastics, Nagano, Japan) with a height of 1.0 mm and an internal diameter of 0.79 mm were placed on each specimen surface. Each tube was carefully filled with either ECII or RXU dual-polymerising resin cements and then light-cured for 40 s. A standard mode (1,000 mW/cm^2^) light-emitting diode (LED) curing unit (Valo, Ultradent, South Jordan, UT, USA) was employed for the curing procedure. Following the light-curing procedure, the micro-bore Tygon tubes were gently detached with a sharp blade. All specimens were then stored in distilled water at 37 °C for 24 h. The specimens were subsequently subdivided into two groups: without thermocycling (0TC) and with 10,000 thermocycles (10kTC). The 10kTC group specimens underwent thermocycling in an apparatus (K178-08, Tokyo Giken, Tokyo, Japan) equipped with two water baths set to 5 °C and 55 °C, with a 30-s dwell time at each temperature. Each group contained 20 specimens.

For the μSBS test, the specimens were mounted on a universal testing machine (EZ-SX, Shimadzu, Kyoto, Japan) and tested at a crosshead speed of 1 mm/min [10]. If a specimen was fractured before the μSBS test, it was documented as a pretesting failure (ptf) and excluded from the μSBS statistical analysis.

### Failure mode analysis

Following the μSBS test, fractured specimens were microscopically analysed using a laser-based confocal imaging system (VK-X150, Keyence, Osaka, Japan) at 10 × magnification. The resulting fracture patterns were categorised into three distinct types: adhesive failure between the substrates and resin cement, cohesive failure within the resin cement, or mixed failure.

### Statistical analysis

All statistical analyses were conducted using SPSS 27 (IBM, Chicago, IL, USA). The significance level was set at 0.05. As the μSBS test data did not follow a normal distribution or exhibit homogeneity of variances, these variables were analysed using t-tests and Welch's t-tests with Bonferroni correction.

## Results

### μSBS

The μSBS test results are presented in Table 2.

**Table 2:** μSBSs to various substrate in MPa

		enamel	dentin	zirconia	LDS	resin block
BLII/ECII	0TC	19.0 ± 6.1^A^	15.5 ± 3.7^a^	20.2 ± 4.4^b^	17.3 ± 7.1	17.4 ± 5.7^B^
	10kTC	25.8 ± 6.6	17.7 ± 5.1^a^	18.1 ± 4.4^b^	6.8 ± 1.0	11.4 ± 3.1
SBU/RXU	0TC	19.4 ± 8.5^A^	10.3 ± 3.7	15.0 ± 2.9	7.1 ± 2.9	17.6 ± 6.4^B^
	10kTC	2.5 ± 1.0	6.6 ± 3.7	3.0 ± 1.2	1.0 ± 0.4	3.8 ± 0.9

All values are given as the mean ± SD

Within the same adhesive/cement combination, values with the same small superscript letter are not significantly different between 0TC and 10kTC (*p* > 0.05)

Within the same thermal cycling condition, values with the same capital superscript letter are not significantly different between BLII/ECII and SBU/RXU (*p* > 0.05)

In the BLII/ECII group, the mean μSBS values before thermocycling were more than 15 MPa for all substrates. Comparing 0TC and 10kTC, thermocycling significantly increased μSBS for enamel (*p* < 0.05), showed no significant difference for dentin and zirconia (*p* > 0.05), and significantly decreased for LDS and resin block (*p* < 0.05). In the SBU/RXU group, the mean μSBS values in 0TC were more than 15 MPa for enamel, zirconia, and resin block. Comparing 0TC and 10kTC, thermocycling significantly decreased μSBS for all the substrates (*p* < 0.05). In the comparison between BLII/ECII and SBU/RXU groups, no significant differences were observed for enamel and resin block at 0TC (*p* > 0.05). However, in the other groups, BLII/ECII demonstrated significantly higher SBS than SBU/RXU (*p* < 0.05).

Table 3 presents the survival percentage from the μSBS test. In the BLII/ECII group, the survival percentage was 100% for enamel and dentin at 10kTC, and for LDS at 0TC and 10kTC. In the SBU/RXU, none of the groups showed a 100% survival percentage, with zirconia at 10kTC showing the lowest survival percentage of 35%.

**Table 3:** Survival percentage of μSBS test (Number of survived specimens/number of pre-test failure specimens)

		enamel	dentin	zirconia	LDS	resin block
BLII/ECII	0TC	95% (19/1)	90% (18/2)	90% (18/2)	100% (20/0)	90% (18/2)
	10kTC	100% (20/0)	100% (20/0)	85% (17/3)	100% (20/0)	85% (17/3)
SBU/RXU	0TC	80% (16/4)	90% (18/2)	65% (13/7)	90% (18/2)	85% (17/3)
	10kTC	50% (10/10)	65% (13/7)	35% (7/13)	45% (9/11)	50% (10/10)

**Fig. 2:**
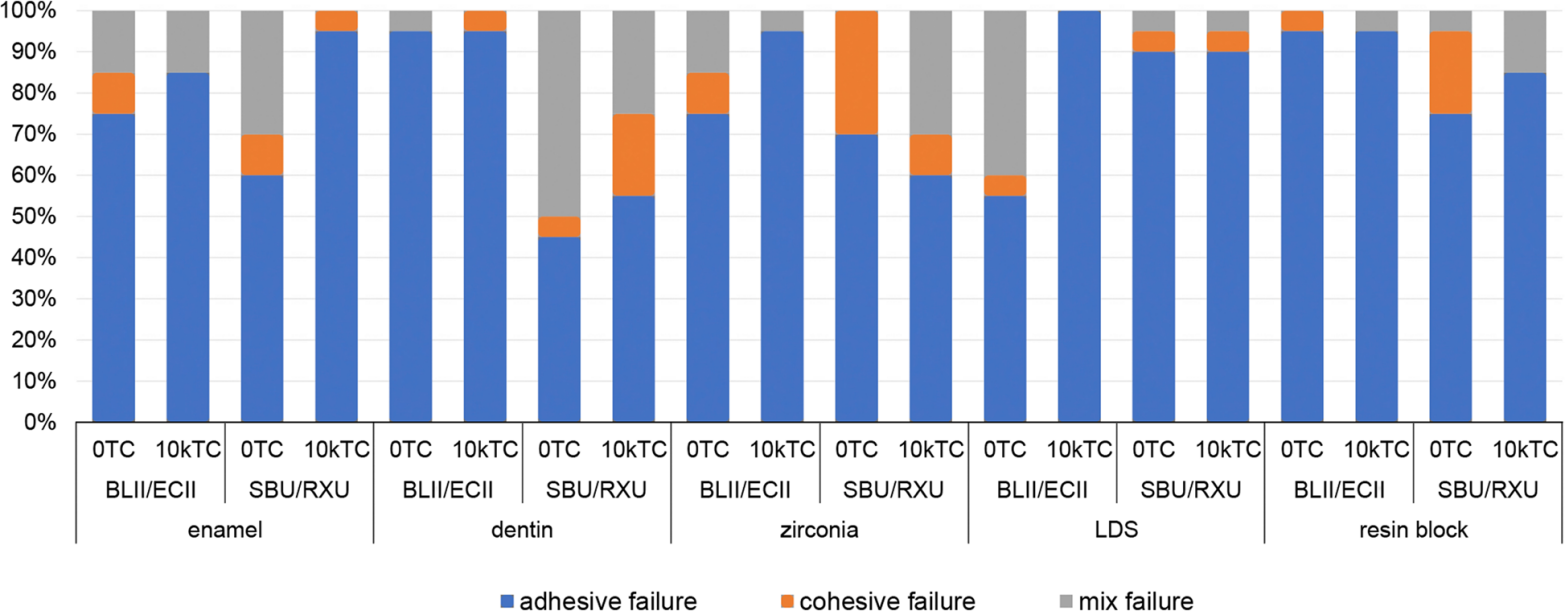
Failure mode distribution

The failure mode analysis results are shown in Fig. 2. Adhesive failure was the most frequently observed failure type across all groups.

## Discussion

BLII and SBU are universal adhesives containing chemical curing initiators that can react with compatible dual-polymerizing resin cements, ECII and RXU, respectively. This feature, often referred to as ‘touch cure’ or ‘contact cure’ technology, facilitates automatic polymerisation when the adhesive and cement components interact [11–14]. The touch cure or contact cure mechanism is designed to enhance the polymerisation process of resin cements in dental applications.

BLII is a dual-bottle, self-curing universal adhesive system that utilises an acidic three-dimensional self-reinforcing (3D-SR) monomer and ‘Bose technology’ (which employs a borate initiator). When the two liquids are combined, a catalyst containing an aryl borate compound reacts with the 3D-SR monomer to produce a borane derivative. This borane derivative is oxidised by a peroxide component in the mixture and creates free radicals that initiate chemical curing of the adhesive [7, 8]. This study demonstrated that adequate μSBSs for enamel, dentin and zirconia to BLII/ECII were achieved through this mechanism even in 10kTC group.

SBU is a one-bottle universal adhesive system that contains the functional monomers 10-methacryloyloxydecyl dihydrogen phosphate (10-MDP). 10-MDP is recognised as the most reliable functional monomer in dentistry, capable of forming chemical bonds with hydroxyapatite in enamel and dentin, resulting in the formation of MDP-Ca salts [15]. This chemical bonding process complements micromechanical adhesion, and the resulting MDP-Ca salts exhibit high stability and resistance to hydrolysis [16]. 10-MDP also demonstrates high adherence to zirconia [17]. However, in this study, the μSBS and survival percentage of the μSBS test decreased for enamel, dentin and zirconia in the SBU/RXU group after 10kTC. One possible explanation for these results is that the effectiveness of 10-MDP in terms of bond strength and longevity was significantly influenced by variations in its purity and concentration across different product formulations [16]. Another possible reason is that, although SBU is a light-curable material, it was not exposed to light curing in this study, which may have impacted the bonding performance of SBU/RXU. A third probable explanation is that RXU exhibited higher water sorption and solubility compared to the other functional monomers containing resin cements, demonstrating elevated values even for this category of materials [18].

For achieving effective adhesion between resin cements and LDS and resin blocks, silane (generally represented by γ-methacryloxypropyl trimethoxy silane (γ-MPTS)), is frequently used in dentistry field. However, previous research has indicated that γ-MPTS can degrade through hydrolysis when stored in acidic monomers such as 10-MDP [19]. The hydrolytic degradation of silane compounds is a complex process and can be affected by various factors such as acidity of the solution, concentration of silane, and the specific molecular structure of the bifunctional silane monomer [20].

As a structural variant of γ-MPTS, γ-methacryloxypropyltriethoxysilane (γ-MPTES) can also be polymerised, allowing it to react with vinyl functional groups present in the monomeric components of adhesive resin formulations [21]. Both BLII and SBU contain γ-MPTES as its silane component. For BLII, acidic monomer and γ-MPTES are stored in separate containers and mixed immediately before use to achieve high storage stability. For SBU, 3-(aminopropyl) triethoxysilane (APTES) is also contained as silane in addition to γ-MPTES. APTES is extensively used in biotechnology and contains an amino-terminal group and reactive alkoxy moieties [22–25]. APTES can form chemical bonds with ceramic surfaces through its silanol groups, produced when its alkoxy groups undergo hydrolysis [25].

Contrary to our expectations, in this study, LDS and resin blocks in the BLII/ECII and SBU/RXU groups exhibited low μSBSs values after thermocycling.　Previous studies have reported that LDS requires a specific adhesive protocol for optimal clinical performance [26–28]. The process begins with the application of hydrofluoric acid to etch the restoration surface. This etching creates microscopic irregularities that enhance mechanical retention of the composite cement [28]. Following etching, a silane coupling agent is applied to facilitate chemical bonding between the ceramic and the cement, significantly enhancing the long-term durability of the adhesive interface [26]. However, in this study, BLII/ECII group showed a 100% survival percentage for LDS at both 0TC and 10kTC, even in the absence of hydrofluoric acid.

Resin blocks are composed of filler and matrix resin. Studies have suggested that using a resin primer containing methyl methacrylate (MMA) or a combination of silane and a resin primer containing MMA can improve long-term adhesion durability [29, 30]. The low μSBS values observed after thermocycling might be attributed to the absence of hydrofluoric acid treatment for LDS and the lack of resin primer application for the resin block.

Based on these findings, our proposed null hypotheses can be rejected. BLII/ECII demonstrated stable long-term bond strength to enamel, dentin, and zirconia. Further research is necessary to elucidate the mechanisms involved in the adhesion of universal adhesives and relative resin cements to various substrates.

## Conclusion

The findings from this study indicate that the bonding effectiveness to various dental substrates (including enamel, dentin, zirconia, LDS, and resin blocks) was significantly influenced by the choice of universal adhesive systems in combination with dual-polymerising resin cements. The application of thermocycling further impacted the adhesive efficacy across these different materials. The BLII/ECII might be beneficial for long-term stable bond strengths to enamel, dentin, and zirconia.

## Data Availability

The datasets used and analysed during this study are available from the corresponding author upon reasonable request.

## Abbreviations

LDS: Lithium disilicate ceramics
BLII: Bondmer Lightless II
ECII: Estecem II
SBU: Scotchbond Universal Plus Adhesive
RXU: RelyX Universal Resin Cement
0TC: 0 Thermal cycles
10kTC: 10,000 Thermal cycles
ptf: Pretesting failure
3D-SR: Three-dimensional self-reinforcing
10-MDP: 10-Methacryloyloxydecyl dihydrogen phosphate
γ-MPTS: γ-Methacryloxypropyl trimethoxy silane
γ-MPTES: γ-Methacryloxypropyltriethoxysilane
APTES: 3-(Aminopropyl)triethoxysilane
MMA: Methyl methacrylate
